# Synaptic Mitochondrial Oxidative Stress Contributes to Individual Variability in Age‐Related Cognitive Inflexibility in Mice

**DOI:** 10.1111/acel.70716

**Published:** 2026-09-14

**Authors:** Rui Yamada, Hirotaka Nagai, Chisato Numa, Yunhui Zhu, Midori Nagai, Kohei Ota, Yusuke Kawashima, Nobuhiko Ohno, Tomoyuki Furuyashiki

**Affiliations:** ^1^ Division of Pharmacology, Graduate School of Medicine Kobe University Kobe Japan; ^2^ Department of Anatomy, Division of Histology and Cell Biology, School of Medicine Jichi Medical University Shimotsuke Japan; ^3^ Department of Pharmacology Graduate School of Medical and Dental Sciences, Institute of Science Tokyo Tokyo Japan; ^4^ Department of Applied Genomics Kazusa DNA Research Institute Kisarazu Japan; ^5^ Division of Ultrastructural Research National Institute for Physiological Sciences Okazaki Japan

**Keywords:** cognitive aging, mice, mitochondria, oxidative stress, prefrontal cortex, synapse

## Abstract

Aging is associated with impairments in cognitive flexibility, a key executive function supported by the medial prefrontal cortex (mPFC), yet the biological mechanisms underlying individual variability in age‐related decline remain poorly understood. Here we investigated behavioral, ultrastructural, and proteomic correlates of cognitive inflexibility in mice across aging. Using a touchscreen‐based attentional set‐shifting task, we observed substantial individual variability in cognitive inflexibility among aged C57BL/6J mice. Volume electron microscopy of the mPFC revealed age‐related reductions in synaptic density, but these structural changes did not correlate with cognitive performance. Instead, the proportion of synapses containing presynaptic mitochondria was inversely associated with cognitive flexibility in aged mice. To identify molecular correlates, we performed proteomic profiling of mPFC whole tissue and synaptosome fractions. Proteins associated with individual variability in cognitive inflexibility were largely distinct from those associated with chronological aging. Notably, synaptosomal proteins negatively correlated with cognitive performance were strongly enriched for mitochondrial pathways, including oxidative phosphorylation, mitochondrial translation, and the tricarboxylic acid cycle. Consistent with these findings, the mitochondria‐targeted antioxidant MitoQ improved attentional set‐shifting performance in aged mice without affecting initial learning. Proteomic analyses revealed that MitoQ reduced the abundance of synaptosomal mitochondrial proteins, particularly those involved in mitochondrial apoptotic signaling. Together, these results suggest that synaptic mitochondrial oxidative stress in the mPFC contributes to individual vulnerability to cognitive inflexibility. Targeting synaptic mitochondrial oxidative stress may therefore represent a promising strategy to preserve executive function during aging.

## Graphical abstract

Aged mice showed substantial individual variability in cognitive inflexibility, associated with greater abundance of synapse‐associated mitochondrial proteins in the medial prefrontal cortex. The mitochondria‐targeted antioxidant MitoQ improved attentional set‐shifting performance and reduced the abundance of synaptosomal mitochondrial proteins, supporting synaptic mitochondrial oxidative stress as a potential target during aging.

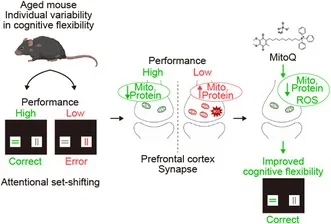

## 1 Introduction

Aging is a complex biological process that affects multiple brain systems and often leads to cognitive decline, compromising independent living and quality of life. In rodents, the biological bases of age‐related impairments in long‐term memory have been studied extensively, whereas the mechanisms underlying age‐related cognitive inflexibility, an executive deficit that also strongly impacts everyday functioning, remain less explored. The prefrontal cortex (PFC) supports higher‐order cognitive functions, including working memory, attention, and cognitive flexibility, and age‐related PFC dysfunction is linked to executive impairments across humans, non‐human primates, and rodents (Murman 2015; West 1996; Burke and Barnes 2006). Across species, aging is associated with dendritic atrophy, reduced synaptic density in PFC neurons, and changes in synaptic molecules (Burke and Barnes 2006; Bloss et al. 2011; Morrison and Baxter 2012). Aging is also linked to altered brain metabolism and increased oxidative stress (Lee and Kim 2022; Floyd and Hensley 2002), changes that could disrupt synaptic integrity and plasticity by perturbing cellular homeostasis and energy balance. However, how these synaptic and molecular alterations in the PFC contribute to age‐related cognitive inflexibility remains unclear. Because cognitive inflexibility varies substantially among aged individuals, identifying the biological mechanisms that underlie this individual variability is important to develop preventive and therapeutic strategies to preserve cognitive flexibility during aging.

Cognitive flexibility and its individual variability are commonly assessed across species using attentional set shifting, a measure of executive control (Rhodes 2004; Heisler et al. 2015; Ianov et al. 2016; Barense et al. 2002; Nagai 2024; Birrell and Brown 2000). This process requires maintaining the relevant rule or stimulus dimension while updating behavior when contingencies change; in aging, impairments often manifest as perseveration and increased susceptibility to interference. In this study, we used an attentional set shifting test to quantify individual variability in age‐related cognitive inflexibility in mice and combined behavioral measures with proteomic analyses of whole tissue and synaptosome fractions from the mPFC. Molecular signatures associated with this variability were distinct from those associated with chronological aging and featured synapse‐specific changes of proteins with diverse functions, especially increased mitochondrial proteins, alongside ultrastructural correlates such as a higher proportion of synapses containing presynaptic mitochondria. Furthermore, the mitochondria‐targeted antioxidant MitoQ remodeled synaptic protein abundance, including reduced pro‐apoptotic mitochondrial proteins, and mitigated cognitive inflexibility in aged mice, supporting a role for mPFC synapse‐associated mitochondrial and redox‐related processes in individual variability in age‐related cognitive inflexibility.

## 2 Results

### 2.1 Individual Variability in Cognitive Inflexibility in Aged C57BL/6J Mice

**FIGURE 1:**
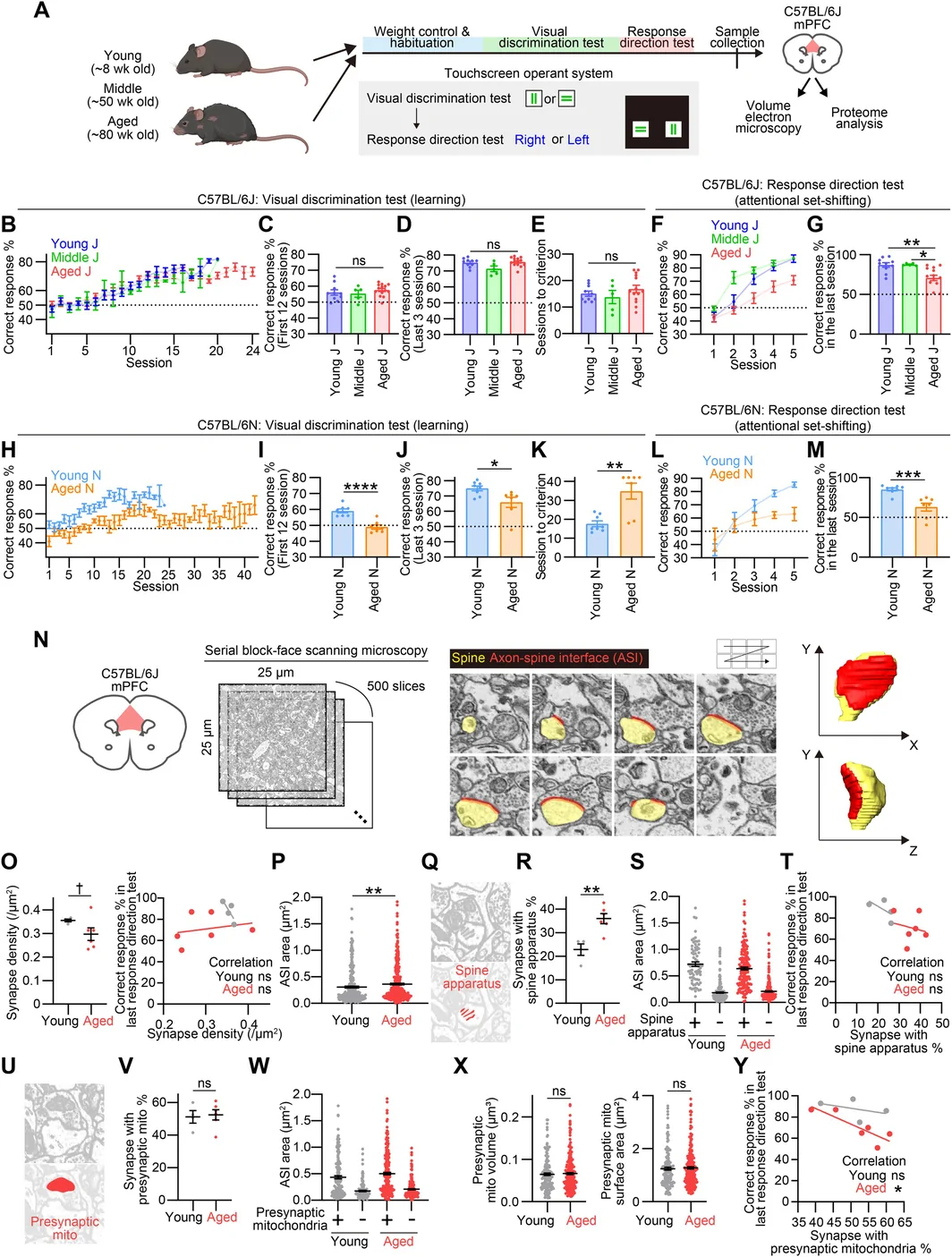
Aged C57BL/6J mice show individual variability in cognitive inflexibility associated with presynaptic mitochondrial abundance in the mPFC. (A) Schematic overview of the touchscreen‐based operant conditioning paradigm. (B–G) Behavioral performance of young, middle‐aged, and aged C57BL/6J mice in the visual discrimination and response direction tests. For the visual discrimination test, the correct response rate averaged within each session throughout the experiment (B), over the first 12 sessions (C), and over the last 3 sessions (D), as well as the number of sessions required to reach the performance criterion (E), are shown. For the response direction test, the correct response rate within each session throughout the experiment (F) and within the last session (G) are shown. Note that the correct response rate in the last session varies considerably among aged C57BL/6J mice. (H–M) Behavioral performance of young and aged C57BL/6N mice in the visual discrimination and response direction tests. For the visual discrimination test, the correct response rate averaged within each session throughout the experiment (H), over the first 12 sessions (I), and over the last 3 sessions (J), as well as the number of sessions required to reach the performance criterion (K), are shown. For the response direction test, the correct response rate within each session throughout the experiment (L) and within the last session (M) are shown. Note that the behavioral performance is uniformly impaired among aged mice in both tests. (N) Schematic illustration of volume electron microscopy used to examine synaptic ultrastructure and schematic of axon‐spine interface (ASI) annotation, a structural proxy for synapse size. (O) (Left) Synapse density. Three regions within one image stack were analyzed per mouse; each data point represents one mouse. (Right) Correlation between synapse density and cognitive performance (measured by the last session of the response direction test). (P) ASI size. (Q) Representative image of a spine apparatus, typically located at the neck of large spine heads. (R) Proportion of synapses with spine heads containing a spine apparatus. (S) ASI size for synapses with or without a spine apparatus. (T) Correlation between the proportion of spine apparatus‐containing synapses and cognitive performance. (U) Representative image of presynaptic mitochondria. (V) Proportion of synapses containing presynaptic mitochondria. (W) ASI size for synapses with or without presynaptic mitochondria. (X) Volume and surface area of presynaptic mitochondria. (Y) Correlation between the proportion of presynaptic mitochondria‐containing synapses and cognitive performance. Scale bars: One edge of each image in (N, Q, U) corresponds to 2 μm. Data are presented as mean ± SEM. Statistical analyses were performed using one‐way ANOVA with Holm‐Sidak post hoc multiple comparisons (C,D,E,G), unpaired two‐tailed t‐test (I–K, M, O, P, R, V, X), and Pearson's correlation test (O, T, Y). †*p* < 0.1, *p < 0.05, **p < 0.01, ***p < 0.001, ****p < 0.0001; ns, not significant. Ten young, five middle‐aged, and 14 aged male C57BL/6J mice were used in (B–G). Eight young and seven aged male C57BL/6N mice were used in (H–M). Four young and six aged male C57BL/6J mice following behavioral tests were used in (N–Y). Each dot in (C–E, G, I–K, M, O, R, T, V, Y) represents an individual mouse. Each dot in (P, S, W, X) represents an individual synapse.

To evaluate cognitive flexibility, we used an attentional set‐shifting test conducted in touchscreen‐based operant chambers (Figure 1A) (Mar et al. 2013). Mice were first trained on a visual discrimination test, in which two distinct visual stimuli (vertical or horizontal lines) were simultaneously presented on a touchscreen. They had to select the predetermined correct stimulus, regardless of its position (left or right), to receive a food reward. After reaching a criterion accuracy, mice advanced to a response‐direction test, where they were required to choose the correct side (left or right), irrespective of the stimulus pattern, to obtain the reward.

While C57BL6 mice are widely used in behavioral research, two substrains, C57BL/6J and C57BL/6N, reportedly show distinct age‐related behavioral abnormalities in exploration, anxiety, and sociability (Yamada et al. 2025). We therefore compared male mice of these substrains across three age groups: young (8–12 weeks), middle‐aged (50 weeks), and aged (75–85 weeks). All C57BL/6J mice successfully learned the visual discrimination phase (Figure 1B–E). In the response‐direction phase, young and middle‐aged mice adapted successfully; however, some aged mice failed, while others performed comparably to young mice, indicating individual variability in age‐related impairment in attentional set shifting ability (Figure 1F,G). Middle‐aged mice were included specifically to determine when this individual variability first emerges; the absence of variability at this intermediate age indicates that it arises specifically in advanced age, rather than progressively across adulthood. In contrast, whereas young C57BL/6N mice correctly performed the visual discrimination and response direction tests, aged C57BL/6N mice showed poor performance in both tests even after extended training, suggesting broader learning deficits (Figure 1H–M). We selected the C57BL/6J strain for the subsequent proteomic and mechanistic experiments because, unlike C57BL/6N mice, aged C57BL/6J mice retained intact visual discrimination learning while showing selective, individually variable impairment specifically in the response‐direction (attentional set‐shifting) phase, making this strain well suited for isolating molecular correlates of individual variability in cognitive inflexibility independent of more general learning impairment. Both male and female C57BL/6J mice successfully learned visual discrimination, although the acquisition rate appeared to be slower in females. In the subsequent response direction test, both male and female C57BL/6J mice showed similar age‐related cognitive inflexibility (Figure S1). Thus, we only examined males for subsequent proteomic and mechanistic experiments in this study.

We examined the correlation between visual discrimination learning‐phase metrics (percent correct over the first 12 sessions, percent correct over the last 3 sessions, and sessions to reach criterion) and response‐direction performance. None of these visual discrimination learning‐phase metrics significantly correlated with response‐direction performance in aged mice (first 12 sessions, *r* = −0.093, *p* = 0.75; last 3 sessions, *r* = 0.126, *p* = 0.67; sessions to criterion, *r* = −0.286, *p* = 0.32; data not shown graphically), indicating that this individual variability is not attributable to differences in general visual discrimination learning ability.

### 2.2 Correlation Between Age‐Related Cognitive Inflexibility and Presynaptic Mitochondrial Abundance

To identify structural correlates of cognitive flexibility, as measured by final behavioral accuracy in the response direction test for attentional set shifting, we performed volume electron microscopy of the mPFC in aged and young mice (Figure 1N). Aged mice showed a reduced density of synapses on dendritic spines (*p* = 0.0799, Hedges' *g* = 1.02), accompanied by a larger average axon‐spine interface (ASI) and a higher proportion of spine apparatus‐containing synapses (*p* = 0.0051, Hedges' *g* = 2.31), which were associated with large ASI areas, suggesting a preferential loss of smaller synapses lacking spine apparatuses (Figure 1O–S). Despite these age‐associated changes in synaptic density and structure, none of these parameters correlated with individual variability in cognitive performance (Figure 1O,T). Astrocytic coverage of synapses, a component of the tripartite synapse, did not differ significantly between young and aged mice, as measured by the proportion of synaptic perimeters covered by astrocytic processes (19.34% ± 2.27% and 16.93% ± 1.45%, respectively; *p* = 0.35, Student's *t*‐test), nor did it correlate with cognitive performance in aged mice (Pearson's correlation coefficient, *r* = 0.146; *p* = 0.7826; data not shown graphically). Notably, presynaptic mitochondria, typically observed in presynaptic terminals apposing dendritic spines with larger ASIs, showed a distinct relationship to age‐related cognitive inflexibility. Although neither the overall frequency (*p* = 0.8029, Hedges' *g* = 0.15) nor morphology of presynaptic mitochondria, nor their association with ASI size, changed significantly with age (Figure 1U–X), the proportion of synapses containing presynaptic mitochondria was inversely correlated with cognitive performance (i.e., positively associated with cognitive inflexibility) in aged but not young mice (Figure 1Y). These findings suggest that reduced synaptic density in the mPFC of aged mice is not, by itself, sufficient to explain cognitive inflexibility, and that differences in presynaptic mitochondrial abundance may contribute to this individual variability.

### 2.3 Distinct mPFC Proteomic Signatures of Aging and Cognitive Inflexibility

**FIGURE 2:**
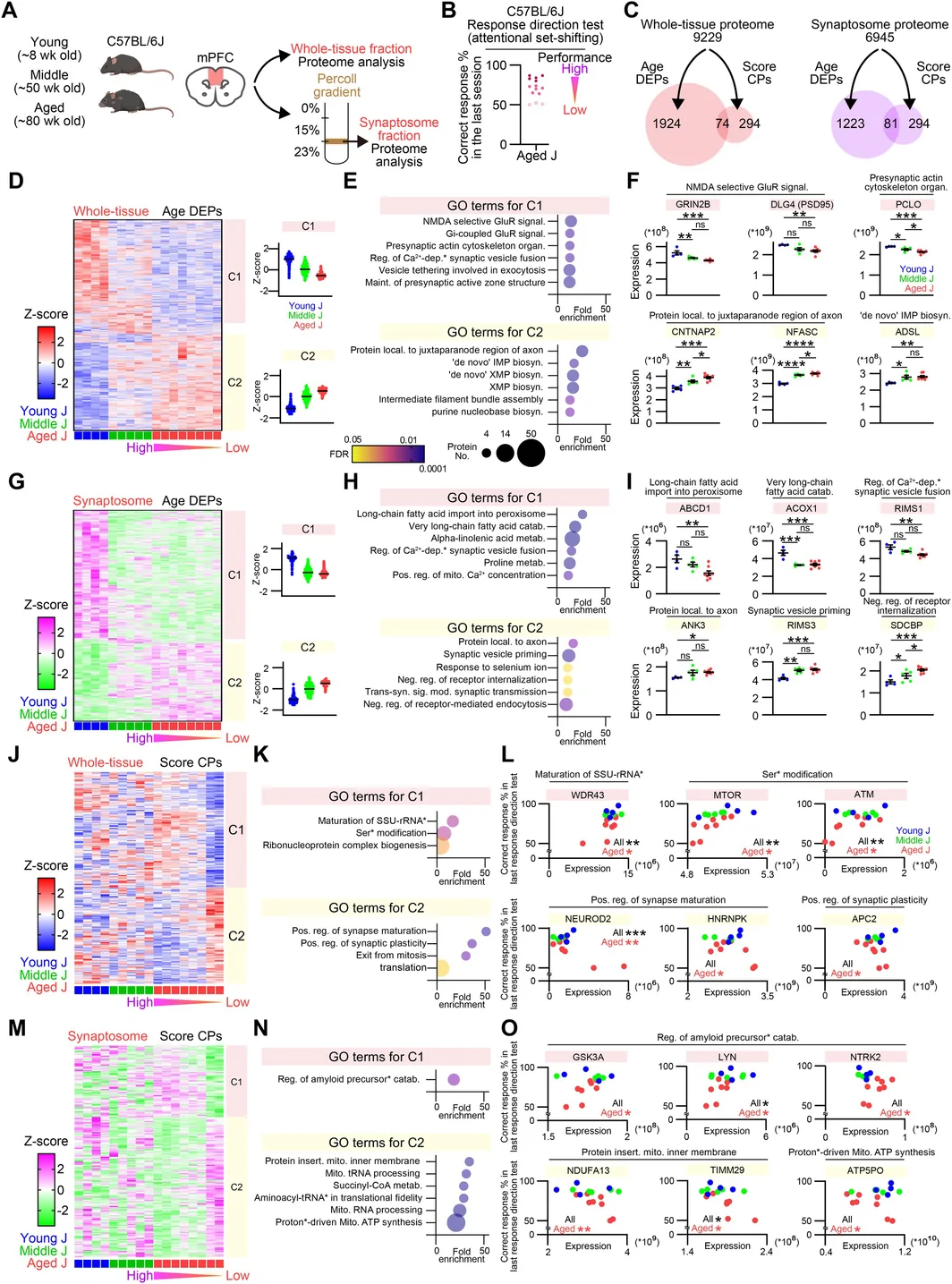
The mPFC proteome reveals distinct molecular signatures associated with chronological aging and individual variability in cognitive inflexibility. (A) Schematic of sample preparation for proteomic analyses. Whole tissue and synaptosome fractions were isolated from the mPFC of male C57BL/6J mice across different age groups using a density gradient centrifugation method. (B) Cognitive performance during the last session of the response direction test in aged C57BL/6J mice. Data from aged mice in Figure 1G are shown again to illustrate individual variability in cognitive inflexibility. (C) Age DEPs and Score CPs identified in whole tissue and synaptosome fractions (see Methods), with the overlap between Age DEPs and Score CPs at the chance level. (D–I) Clustering analysis of Age DEPs in whole tissue (D–F) or synaptosome (G–I) samples, identifying two clusters, Cluster 1 (downregulated in aged mice) and Cluster 2 (upregulated in aged mice) (D, G), their enriched GO terms (E, H), and the expression of representative proteins (F, I). (J–O) Clustering analysis of Score CPs in whole tissue (J–L) or synaptosome (M–O) samples, identifying two clusters, Cluster 1 (downregulated in aged mice with greater decline in cognitive flexibility) and Cluster 2 (upregulated in aged mice with greater decline in cognitive flexibility) (J, M), their enriched GO terms (K, N), and the expression of representative proteins (L, O). signal.; signaling pathway, dep.*; dependent activation of, maint.; maintenance, local.; localization, biosyn.; biosynthetic process, reg.; regulation, pos.; positive, metab.; metabolic process, mito.; mitochondrial, neg.; negative. Data are presented as mean ± SEM. Statistical analyses were performed using one‐way ANOVA with Holm‐Sidak post hoc multiple comparisons (F, I) and Pearson's correlation test (L, O). *p < 0.05, **p < 0.01, ***p < 0.001, ****p < 0.0001; ns, not significant. Four young, five middle‐aged, and eight aged male C57BL/6J mice were used following behavioral tests. Each column in (D, G, J, M) and each dot in (F, I, L, O) represents an individual mouse.

To identify molecular correlates of age‐related cognitive inflexibility, we performed proteomic profiling of whole‐tissue and synaptosome fractions in the mPFC, identifying proteins whose abundance varied across age groups (young, middle‐aged, and aged) and correlated with attentional set shifting performance in aged mice (hereafter Age DEPs and Score CPs, respectively; DEPs, differentially expressed proteins; CPs, correlated proteins) (Figure 2A–C). Score CPs were defined using a nominal (uncorrected) *p* < 0.05 threshold (368 and 375 proteins in whole tissue and synaptosome, respectively) and were considered exploratory, as they did not reach statistical significance following Benjamini‐Hochberg FDR correction, reflecting the limited statistical power afforded by the small sample size. To validate the robustness of these correlations, we additionally performed an exact permutation test (see Methods), which yielded a similar number of proteins reaching *p* < 0.05 (378 and 383 in whole tissue and synaptosome, respectively), with 95.7% and 95.5% of the nominally significant proteins, respectively, also reaching *p* < 0.05 under the permutation‐based test; permutation‐based *p* values closely matched the parametric Pearson *p* values across all tested proteins (*r* = 0.997 and 0.998 for whole tissue and synaptosome, respectively), confirming that the nominal correlations are numerically robust and not an artifact of the parametric approximation. To evaluate the biological functions of these CPs without first selecting a subset of statistically significant proteins, we performed rank‐based gene set enrichment analysis (GSEA), ranking all proteins by their signed correlation coefficient with cognitive performance. This threshold‐free analysis identified significant pathway‐level enrichment (FDR *q* < 0.05) of mitochondrial and oxidative phosphorylation‐related Gene Ontology terms (Figure S2), consistent with the GO analysis of nominal Score CPs described further below. Notably, the overlap between Age DEPs and Score CPs was at the chance level (expected vs. observed overla*p* = 76.7 proteins vs. 74 proteins for whole tissue, *p* = 0.61; 70.4 proteins vs. 81 proteins for synaptosomes, *p* = 0.23; Fisher's exact test), suggesting that neural mechanisms determining individual variability in cognitive inflexibility are distinct from chronological aging processes.

To gain functional insights into Age DEPs and Score CPs, we performed gene ontology (GO) analysis. In whole tissue Age DEPs, those decreasing with age were associated with NMDA receptor‐mediated signaling, including GRIN2B and DLG4 (PSD95), and presynaptic actin cytoskeleton organization, including PCLO, whereas those increasing with age were associated with protein localization to the juxtaparanodal region and de novo inosine monophosphate (IMP) biosynthesis (Figure 2D–F). In synaptosomal Age DEPs, those decreasing with age were associated with peroxisomal fatty acid catabolism, including ABCD1‐3 and ACOX1, whereas those increasing with age were associated with axonal protein localization and synaptic vesicle priming (Figure 2G–I). Thus, distinct age‐related proteomic changes occurred in whole tissue and synaptosome fractions of the mPFC. In whole‐tissue Score CPs, proteins positively correlated with cognitive performance were associated with small subunit (SSU) rRNA maturation and serine/threonine kinase signaling, including MTOR and ATM, whereas those negatively correlated with cognitive performance were associated with positive regulation of synapse maturation and plasticity (Figure 2J–L). In synaptosomal Score CPs, proteins positively correlated with cognitive performance were associated with regulation of amyloid precursor protein catabolism, including GSK3A, LYN and NTRK2 (TrkB), whereas those negatively correlated were associated with mitochondrial processes, including mitochondrial protein regulation and metabolism (Figure 2M–O).

### 2.4 Higher Abundance of Synapse‐Associated Mitochondrial Proteins in Aged Mice With Greater Cognitive Inflexibility

**FIGURE 3:**
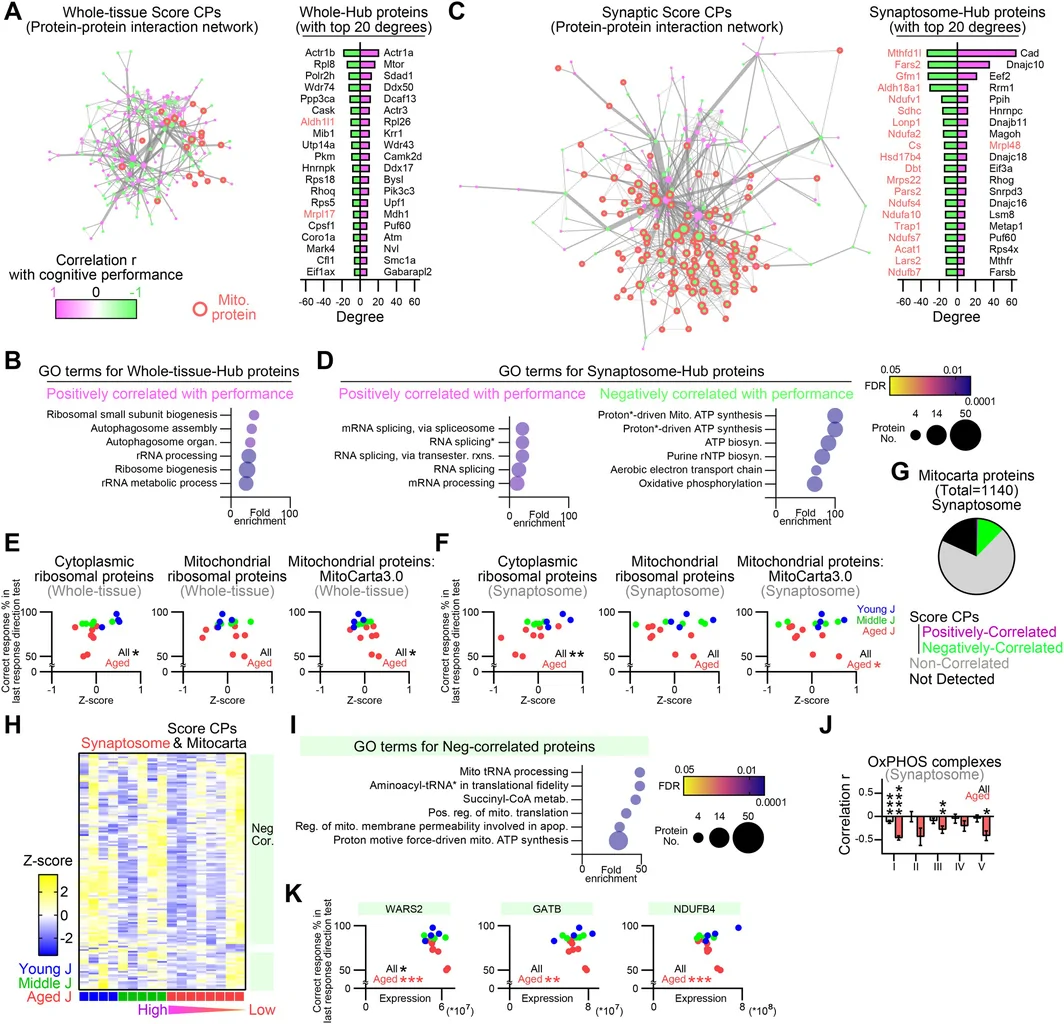
Synapse‐associated mitochondrial proteins are more abundant in aged mice with greater decline in cognitive flexibility. (A–D) Protein–protein interaction networks and the top 20 hub proteins ranked by degrees (number of connections for each node) for Score CPs in whole tissue (A) and synaptosome (C) fractions, along with the GO terms associated with these hub proteins (B and D, respectively). organ.; organization, splicing*; splicing, via transesterification reactions with bulged adenosine as nucleophile, transester.; transesterification, rxns.; reactions, proton*; proton motive force, biosyn.; biosynthetic process. (E,F) Correlations between the expression levels of cytoplasmic or mitochondrial ribosomal proteins, or total mitochondrial proteins in whole tissue (E) and synaptosome (F) fractions and cognitive performance (measured by the last session of the response direction test). (G) Proportions of MitoCarta‐listed proteins (*n* = 1140) that were positively correlated with cognitive performance (Positively‐correlated), negatively correlated with cognitive performance (Negatively‐correlated), not correlated with cognitive performance (Non‐correlated), or not detected in synaptosome fractions. Note that a fraction of the positively correlated proteins in the pie chart is not visible due to their small number. (H) Clustering analysis of MitoCarta‐listed Score CPs, most of which were negatively correlated with cognitive performance. (I) GO terms for Mitocarta‐listed Score CPs negatively correlated with cognitive performance. (J) Average correlation coefficients between the expression levels of proteins in each respiratory chain complex and cognitive performance. (K) Expression levels of representative proteins that are negatively correlated with cognitive performance. Statistical analyses were performed using Pearson's correlation tests (E, F, J, K) and using one‐sample *t*‐test against the chance level (0) in (J). Data are presented as mean ± SEM. *p < 0.05, **p < 0.01, ***p < 0.001, ****p < 0.0001. Four young, five middle‐aged, and eight aged male C57BL/6J mice were used following behavioral tests. Each column in (H) and each dot in (E, F, K) represents an individual mouse.

To identify the core protein signatures underlying individual variability in age‐related cognitive inflexibility, we examined the protein–protein interaction network of Score CPs using the STRING database and identified hub proteins based on degrees (number of connections for each node). In the whole‐tissue proteome, hub proteins positively correlated with cognitive performance were associated with ribosomal and autophagosomal regulation (Figure 3A,B). In contrast, in the synaptosomal proteome, hub proteins positively correlated with cognitive performance were associated with mRNA splicing pathways, including HNRNPC, a splicing factor that also regulates mRNA metabolism and ribonucleoprotein assembly in the cytoplasm (Cieniková et al. 2015), whereas those negatively correlated with cognitive performance were mostly associated with mitochondrial functions, especially mitochondrial oxidative phosphorylation (Figure 3C,D).

Because the interaction network of synaptosomal Score CPs was dominated by mitochondrial proteins, we focused subsequent analyses on mitochondrial proteins. In aged mice, mitochondrial proteins listed in the MitoCarta3.0 database (Rath et al. 2021), especially in synaptosomes, showed negative correlations with cognitive performance (Figure 3E,F), which were absent in young and middle‐aged mice. Although synaptic mitochondria are thought to reside mainly in presynaptic terminals (Brown et al. 2006), synaptosome preparations contain a mixture of pre‐ and postsynaptic and glial components (Figure S3). We therefore refer to the mitochondrial proteins detected in this fraction as synapse‐associated, rather than exclusively presynaptic. These findings thus indicate an association between synapse‐associated mitochondrial protein abundance and age‐related cognitive inflexibility. Conversely, cytoplasmic ribosomal proteins in synaptosomes were positively correlated with cognitive performance. Such changes observed in synaptosomes were marginal in the whole tissue.

Consistent with the negative correlation between MitoCarta‐listed synaptosomal proteins overall and cognitive performance, we identified a subset of mitochondrial proteins in synaptosomes (135/1140) that were negatively correlated with cognitive performance, whereas only a few (5/1140) were positively correlated (Figure 3G,H). GO analysis showed that proteins negatively correlated with cognitive performance were associated with mitochondrial translation, the TCA cycle, and oxidative phosphorylation (Figure 3I,J), as exemplified by several representative proteins (Figure 3K).

Collectively, these findings indicate that synapse‐associated mitochondrial proteins in the mPFC are more abundant in aged mice with greater cognitive inflexibility.

### 2.5 MitoQ‐Mediated Prevention of Age‐Related Cognitive Inflexibility, Associated With Reduced Proapoptotic Proteins in Synapse‐Associated Mitochondria

**FIGURE 4:**
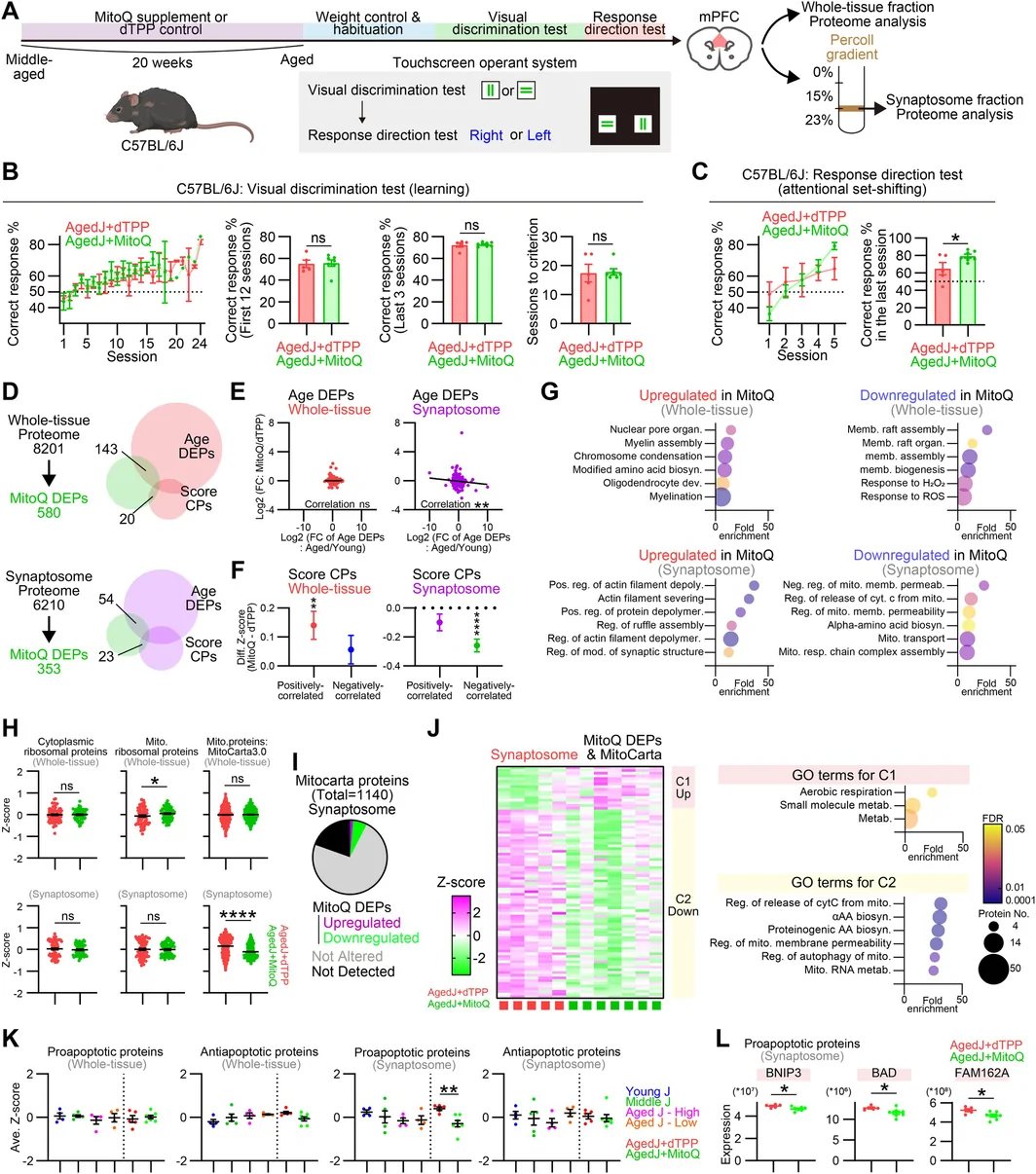
Treatment with the mitochondria‐targeted antioxidant MitoQ reduces synapse‐associated mitochondrial protein abundance, including pro‐apoptotic proteins, and mitigates age‐related cognitive inflexibility. (A) Experimental timeline. C57BL/6J mice were administered either 100 μM MitoQ or dTPP (control) via drinking water from 55 to 75 weeks of age (20 weeks) and throughout the subsequent behavioral experiments, followed by proteomic analyses of whole tissue and synaptosome fractions of the mPFC. (B, C) Behavioral performance of MitoQ‐ or dTPP‐treated mice in the visual discrimination and response direction tests. For the visual discrimination test, the correct response rate averaged within each session throughout the experiment, over the first 12 sessions, and over the last 3 sessions, as well as the number of sessions required to reach the performance criterion, are shown (B). For the response direction test, the correct response rate within each session throughout the experiment and within the last session are shown (C). (D) Venn diagrams of MitoQ DEPs, Age DEPs, and Score CPs in whole tissue and synaptosome fractions. (E) Correlations between age‐associated changes (aged versus young mice) and MitoQ‐induced changes (MitoQ‐ versus dTPP‐treated mice) of Age DEPs in whole tissue and synaptosome fractions. (F) MitoQ‐induced changes in the expression levels of Score CPs in whole tissue and synaptosome fractions that are positively or negatively correlated with cognitive performance. (G) GO analysis of MitoQ DEPs of whole tissue and synaptosome fractions. biosyn.; biosynthetic process, dev.; development, memb.; membrane, organ.; organization, pos.; positive, reg.; regulation, depolymer.; depolymerization, mod.; modification, neg.; negative, cyt.; cytochrome, mito.; mitochondria, resp.; respiratory. (H) Expression levels of cytoplasmic (left) and mitochondrial (middle) ribosomal proteins, and total mitochondrial proteins defined by the MitoCarta3.0 database (right) in whole tissue (upper) and synaptosome (lower) fractions. (I) Proportions of MitoCarta‐listed proteins (*n* = 1140) whose expression levels were upregulated, downregulated, not altered, or not detected in synaptosome fractions. Note that a fraction of upregulated proteins in the pie chart is not visible due to their small number. (J) Clustering analysis of MitoCarta‐listed MitoQ DEPs, identifying two clusters, Cluster 1 (upregulated after MitoQ treatment) and Cluster 2 (downregulated after MitoQ treatment), and their enriched GO terms. (K) Averaged expression levels of pro‐ and anti‐apoptotic proteins across age groups and in MitoQ‐treated mice. (L) Expression of representative pro‐apoptotic proteins in synaptosomes from MitoQ‐ and dTPP‐treated aged mice. Data are presented as mean ± SEM. Statistical analyses were performed using unpaired two‐tailed *t*‐test (B, C, H, K, L), Pearson's correlation test (E) and one‐sample *t*‐test (F). *p < 0.05, **p < 0.01, ***p < 0.001, ****p < 0.0001; ns, not significant. Five and seven mice that received drinking water containing dTPP and MitoQ, respectively, were used. Each dot in (B, C, K, L) represents an individual mouse.

Given the negative correlations of the synapse‐associated mitochondrial proteome with cognitive performance in aged mice, we hypothesized that synapse‐associated mitochondrial dysfunction might underlie age‐related cognitive inflexibility. To test this hypothesis, we assessed the behavioral effects of MitoQ, a mitochondria‐targeted antioxidant with beneficial effects in animal models of various diseases involving mitochondrial dysfunction (McManus et al. 2011; Young and Franklin 2019; Solesio et al. 2013). Systemic MitoQ, but not its inactive analog dTPP, administered for 20 weeks significantly improved attentional set shifting (i.e., the response direction test; *p* = 0.0496, Hedges' *g* = 1.21) in aged mice without altering preceding visual discrimination learning (first 12 sessions, *p* = 0.8822, Hedges' *g* = 0.08; last 3 sessions, *p* = 0.7911, Hedges' *g* = 0.15; sessions to criterion, *p* = 0.8750, Hedges' *g* = 0.09) (Figure 4A–C), implicating mitochondrial dysfunction in age‐related cognitive inflexibility.

To investigate underlying mechanisms, we profiled MitoQ‐regulated proteins (MitoQ DEPs) in mPFC whole tissue and synaptosomes. MitoQ DEPs partially overlapped with both Age DEPs and Score CPs (Figure 4D). In synaptosomes, but not in whole tissue, age‐associated changes were negatively correlated with MitoQ‐induced changes, indicating that MitoQ counteracted age‐related alterations in the synaptosomal proteome, at least partly (Figure 4E). In addition, MitoQ upregulated Score CPs positively correlated with cognitive performance in whole tissue, while downregulating Score CPs negatively correlated with performance in synaptosomes (Figure 4F). Together, these results implicate MitoQ‐sensitive mitochondrial dysfunction in both chronological aging and individual variability in cognitive inflexibility.

GO analysis showed that synaptosomal proteins downregulated by MitoQ were enriched for mitochondrial apoptotic processes, including negative regulation of mitochondrial membrane permeabilization and cytochrome c release (Figure 4G). Beyond mitochondrial processes, GO terms indicated the effects of MitoQ across compartments: in synaptosomes, MitoQ increased actin‐reorganization factors (e.g., cofilin and ADF); in whole tissue, it increased proteins involved in nuclear organization and myelination; and it decreased proteins linked to membrane raft assembly and responses to ROS, consistent with MitoQ's antioxidant action. These findings indicate that MitoQ induces broad proteomic remodeling across subcellular compartments.

Because synaptosomal mitochondrial proteins were negatively correlated with cognitive performance, whereas cytoplasmic ribosomal proteins were positively correlated, as described (see Figure 3E,F), we examined whether MitoQ affected these protein groups. MitoQ selectively decreased MitoCarta‐listed mitochondrial proteins in synaptosomes, with little effect in whole tissue (Figure 4H). However, mitochondrial ribosomal proteins in synaptosomes were unchanged, suggesting that MitoQ targets other mitochondrial pathways. Cytoplasmic ribosomal proteins in synaptosomes were also unaffected.

Consistent with the overall downregulation, a subset of mitochondrial proteins in synaptosomes (70/1140) was downregulated by MitoQ, whereas fewer (16/1140) were upregulated (Figure 4I,J). GO analysis associated the downregulated proteins with the mitochondrial apoptotic pathway (i.e., regulation of release of cytochrome c and mitochondrial membrane permeability), whereas the upregulated proteins were linked to aerobic respiration (Figure 4J). Notably, among apoptosis‐related mitochondrial proteins, MitoQ selectively decreased pro‐apoptotic (*p* = 0.0041, Hedges' *g* = 2.00), but not anti‐apoptotic (*p* = 0.6792, Hedges' *g* = 0.23), proteins in synaptosomes (Figure 4K,L).

Together, these data demonstrate that synapse‐associated mitochondrial dysfunction contributes to individual variability in age‐related cognitive inflexibility and can be ameliorated by a mitochondria‐targeted antioxidant, possibly through multiple downstream pathways, including mitochondria‐derived apoptosis signaling.

## 3 Discussion

Aging is associated with cognitive inflexibility, yet its severity varies markedly among individuals and the underlying biological mechanisms remain poorly understood. In this study, we combined proteomic analyses of the mPFC together with behavioral assessments of attentional set shifting in mice across different age groups. Proteomic profiling of synaptosomes and whole tissue fractions showed that molecular correlates of individual variability in cognitive inflexibility are distinct from those associated with chronological aging, each reflected by unique protein‐expression patterns. Notably, mice exhibiting greater cognitive inflexibility showed synapse‐specific changes in proteins with diverse functions, especially an upregulation of mitochondrial proteins, together with an increased proportion of synapses containing presynaptic mitochondria. The role of synapse‐associated mitochondrial dysfunction was further supported by the observation that the mitochondria‐targeted antioxidant MitoQ mitigated cognitive inflexibility, along with a selective reduction in a subset of synapse‐associated mitochondrial proteins, particularly proapoptotic proteins. Collectively, synapse‐associated mitochondrial dysfunction in the mPFC, distinct from chronological age‐related processes, emerges as a key determinant of individual variability in cognitive aging and offers a potential target for prevention and intervention.

A key insight from our work is that synapse‐associated mitochondria constitute a latent vulnerability that interacts with chronological age‐related processes to cause cognitive inflexibility, possibly through their ROS production, which is directly targeted by MitoQ. Although presynaptic mitochondrial abundance is not broadly altered by chronological age, it varies markedly among individuals even at young ages. Higher mitochondrial abundance predicts age‐related cognitive inflexibility; however, this relationship becomes evident only after aging. Consistent with this developmental trajectory, middle‐aged C57BL/6J mice showed no individual variability in cognitive inflexibility, indicating that this behavioral heterogeneity, like its underlying presynaptic mitochondrial vulnerability, manifests specifically with advanced age rather than earlier in adulthood. In aged mice with greater cognitive inflexibility, synapse‐associated mitochondria show elevated expression of oxidative phosphorylation proteins, which may in turn increase mitochondrial ROS production. Electron microscopy directly identifies presynaptic mitochondria, whereas synaptosome preparations contain a mixture of pre‐ and postsynaptic and glial components. Consequently, the synaptosomal proteome does not selectively reflect presynaptic mitochondria. To establish conclusions specific to presynaptic mitochondria, it will be necessary to isolate and analyze a presynaptic‐enriched mitochondrial fraction, ideally from the same cohort examined by electron microscopy. Because synapse‐associated mitochondrial tRNA‐related proteins are also increased, this upregulation may underlie higher mitochondrial abundance at synapses, accompanied by elevated expression of oxidative phosphorylation proteins and increased ROS production. fMRI studies have reported increased mPFC activity in aged individuals (Nyberg et al. 2012; Morcom and Henson 2018), and such hyperactivity could also enhance mitochondrial ROS generation via Ca^2+^ entry into mitochondria (Feissner et al. 2009). Because peroxisomes mitigate mitochondrial oxidative stress through contact‐mediated ROS transfer (DiGiovanni et al. 2025), age‐associated downregulation of synaptic peroxisome functions, indicated by decreased peroxisomal proteins such as ABCD1‐3 and ACOX1, could permit mitochondrial ROS to exert harmful effects.

Although the precise mechanisms linking synapse‐associated mitochondrial ROS to cognitive inflexibility remain unresolved, synaptic proteomics with MitoQ implicate multiple pathways. For example, pro‐apoptotic mitochondrial proteins, such as BNIP3, BAD, and FAM162A, may mediate ROS's deleterious effects (Orrenius et al. 2007), as MitoQ reduced their synaptosomal levels along with its pro‐cognitive effect. We note that BNIP3, BAD, and FAM162A were classified as pro‐apoptotic based on their reported functions in the literature; we did not directly assess apoptotic signaling (e.g., caspase‐3 activation) in this study, and future work will be needed to confirm whether these mitochondrial protein changes are accompanied by active apoptotic signaling at synapses. Synaptic apoptosis signaling mediated by caspase‐3 reportedly triggers microglial phagocytosis of synapses via complement cascades in cultured neurons (Andoh et al. 2025). In addition, since MitoQ upregulated several cytoskeletal regulators, including ADF/cofilin, mitochondrial ROS could also perturb synaptic actin dynamics, which are critical for synaptic function and plasticity (Kovaleva et al. 2025).

Whereas the present study found that increased abundance of mitochondrial electron transport chain components in mPFC synaptosomes was associated with cognitive impairment in aged C57BL/6J mice, potentially through increased mitochondrial ROS production, previous studies reported that aged C57BL/6N mice with impaired reversal learning exhibited reduced expression of mitochondrial electron transport chain genes and decreased oxidative phosphorylation, along with reactive gliosis, in whole hippocampal tissue (Logan et al. 2023; Baier et al. 2025). This apparent discrepancy may reflect differences in the tissue compartment analyzed (whole tissue versus synaptosomes), the brain region examined (hippocampus versus mPFC), the genetic background and age of the mice, or the specific cognitive domain assessed. Nevertheless, these findings suggest that mitochondria may exert either beneficial or detrimental effects during cognitive aging. They therefore underscore the importance of determining how aging affects distinct mitochondrial populations across brain regions and subcellular compartments, and how these populations influence cognitive function in aged mice.

Several additional limitations should be noted. First, given the modest sample sizes in the MitoQ behavioral cohort (dTPP, *n* = 5; MitoQ, *n* = 7), these findings should be interpreted with appropriate caution pending replication in a larger cohort. Second, dTPP was used as a vehicle control for MitoQ in this study, although dTPP itself might have some effects. However, dTPP has been widely used and validated as a vehicle control for evaluating the antioxidant effects of MitoQ, because it lacks the antioxidant ubiquinone moiety while retaining the lipophilic triphenylphosphonium cation that drives mitochondrial accumulation of MitoQ. Indeed, the behavioral performance of dTPP‐treated aged mice in the response direction test (range, 44%–81%; mean, 64.8%) closely overlapped with that of our original untreated aged cohort (range, 50%–84%; mean, 71.0%), suggesting that dTPP did not exert a marked effect on cognitive performance in aged mice. Because the proteomic analyses in this study directly compared MitoQ‐ and dTPP‐treated aged mice, the MitoQ‐associated proteomic changes can reasonably be attributed to the antioxidant ubiquinone moiety of MitoQ rather than to the triphenylphosphonium moiety shared by the two compounds.

In conclusion, synapse‐associated mitochondria are critical determinants of individual variability in age‐related cognitive inflexibility. Their harmful influence likely reflects oxidative stress that arises when age‐related factors, such as dysregulated neuronal activity and peroxisome impairment, synergize with pre‐existing vulnerabilities from youth, including higher abundance of synapse‐associated mitochondria with elevated expression of oxidative phosphorylation. This oxidative stress could then engage multiple downstream pathways, including release of mitochondrial proapoptotic proteins and cytoskeletal reorganization, ultimately leading to cognitive inflexibility. These synapse‐associated mitochondria‐centered mechanisms identified in this study offer a promising target for prevention and intervention for cognitive aging. Since dysfunctional mitochondria are also implicated in age‐related neurodegenerative diseases, including dementia, future studies should test whether synaptic mitochondrial oxidative stress also contributes to cognitive inflexibility associated with these diseases.

## 4 Methods

### 4.1 Animals

Male and female C57BL/6J mice were obtained from Jackson Laboratory Japan (Yokohama, Japan), and male and female C57BL/6N mice were purchased from Japan SLC (Shizuoka, Japan). Mice were categorized by age as follows: young (8–12 weeks), middle‐aged (50 weeks), and aged (75–85 weeks). All animals were housed in a controlled facility under stable temperature and humidity conditions with a 12‐h light/12‐h dark cycle. Food and water were provided ad libitum throughout the study, except when food deprivation was required for behavioral testing. Initially, mice were group‐housed (4–5 per cage) and were transitioned to single housing prior to behavioral testing.

All experimental procedures were designed to minimize animal suffering and to reduce the number of animals used, in accordance with the principles of the 3Rs (Replacement, Reduction, and Refinement). Animal health and welfare were monitored daily, and humane endpoints were applied when necessary. All procedures were conducted in accordance with the ARRIVE guidelines and the NIH Guide for the Care and Use of Laboratory Animals and were approved by the Animal Care and Use Committees of Kobe University Graduate School of Medicine.

### 4.2 Behavioral Experiments

Prior to behavioral testing, mice were food‐restricted to approximately 80% of their baseline body weight to enhance motivation for food rewards. All behavioral tests were conducted in operant conditioning chambers equipped with a touchscreen interface and an integrated food tray connected to an automatic pellet dispenser. Mice were first habituated to the operant chamber environment. During this phase, five food pellets (10 mg each) were placed directly on the food tray before the session, and mice were allowed to freely consume them. If any pellets remained uneaten, the same habituation procedure was repeated the following day. Once a mouse consumed all five pellets, it proceeded to the next training phase. To familiarize mice with the food dispenser mechanism, pellets were delivered automatically every 10 s over a 15‐min period, totaling approximately 90 pellets. Mice were then allowed an additional 45 min to consume the pellets, resulting in a 60‐min session. If pellets were left uneaten, the same procedure was repeated the next day to ensure proper acclimatization. In the next phase, visual stimuli consisting of horizontal or vertical lines were displayed on either side of the touchscreen. Touching either side of the screen with the nose resulted in the delivery of a food pellet. Each session lasted for 1 h or until the mouse reached 150 screen touches, whichever occurred first. Mice that performed fewer than 30 nose pokes were re‐exposed to the same test on the following day.

Following touchscreen habituation, mice underwent a visual discrimination test in which they were required to touch a specific stimulus (horizontal or vertical lines) to receive a food reward. Sessions lasted 1 h or until 100 screen touches were reached, whichever occurred first, and mice completed two sessions per day. Mice continued training daily until reaching the predetermined performance criteria: achieving at least 80% correct responses in a single session or more than 75% correct responses in at least two sessions over eight training days. If the performance criteria were not met, training was terminated after 42 sessions. Because the number of sessions required to reach the performance criterion varied among individual mice, the “first 12 sessions” and “last 3 sessions” windows used to summarize visual discrimination performance could overlap for mice that reached criterion early. To assess the attentional set shifting ability, mice then underwent a response direction test, where they were required to touch a specific side of the screen (left or right), irrespective of the visual stimulus, to receive a reward. Each session lasted 1 h or until 100 screen touches were reached, and mice completed one session daily for five consecutive days. Two aged male C57BL/6N mice and one aged male C57BL/6J mouse were excluded from analysis because they performed fewer than 10 trials in the visual discrimination test.

For pharmacological experiments, male C57BL/6J mice were provided with drinking water containing either MitoQ (mitoquinone mesylate, 100 μM) or decyl triphenylphosphonium bromide (dTPP) (100 μM), together with β‐cyclodextrin (400 μM), from 55 to 75 weeks and throughout the subsequent behavioral tests. The dosing regimen was based on previous studies (McManus et al. 2011; Young and Franklin 2019). dTPP is structurally identical to MitoQ but lacks the ubiquinone moiety and thus serves as a control compound that retains the lipophilic cationic carrier without antioxidant functionality. Two MitoQ‐treated mice were excluded from analysis because they did not reach the performance criteria described above in the visual discrimination test.

A subset of the behavioral data from aged and young male C57BL/6J mice (visual discrimination and response‐direction test performance, without additional experimental manipulation; Figure 1B–G) is also reported elsewhere (Yamada et al. 2026), where they served as a control condition in an independent study of microglial repopulation.

### 4.3 Sample Collection

After the behavioral experiments, mice were placed in their home cages with food and water available ad libitum for 2–3 weeks. mPFC tissues were collected for either proteomic analysis or volume electron microscopy.

For proteomic analysis, mice were deeply anesthetized via intraperitoneal injection of sodium pentobarbital (100 mg/kg; Nacalai Tesque, Kyoto, Japan), followed by transcardial perfusion with ice‐cold saline to remove blood. mPFC tissue from each C57BL/6J mouse was processed individually for proteomic analysis and was not pooled across animals. The mPFC, including the anterior cingulate cortex, prelimbic cortex, and infralimbic cortex, was rapidly dissected in the synaptosome isolation buffer, containing 320 mM sucrose, 5 mM HEPES, 0.05 mM dithiothreitol (DTT), 0.1 mM phenylmethylsulfonyl fluoride, 2 U/mL RNaseOUT, and a complete protease inhibitor cocktail (pH 7.4). Tissue was homogenized using a Dounce homogenizer. A portion of the homogenate was reserved for whole fraction analysis. The remaining homogenate was centrifuged at 1000 × g for 10 min at 4°C to remove nuclei. The supernatant was then centrifuged at 20,400 × g for 10 min at 4°C to eliminate microsomal components. The resulting pellet was resuspended in the synaptosome isolation buffer and layered onto a discontinuous Percoll gradient (15% and 23%). Ultracentrifugation was performed at 31,000 × g for 8 min at 4°C. The synaptosome‐enriched fraction was carefully collected and processed for proteomic analysis (DiGiovanni et al. 2012).

For volume electron microscopy, C57BL/6J mice were anesthetized with sodium pentobarbital and perfused transcardially with pre‐warmed saline (37°C), followed by half Karnovsky fixative containing 2.5% glutaraldehyde, 2% paraformaldehyde, and 2 mM calcium chloride in 0.1 M cacodylate buffer (pH 7.4). Brains were removed and post‐fixed overnight in the same fixative at 4°C. The following day, 120 μm‐thick coronal sections were prepared using a vibratome in the cutting solution (0.1 M cacodylate buffer, pH 7.4, with 2 mM calcium chloride). Brain slices were stored at −30°C in cryoprotectant until further processing.

### 4.4 Volume Electron Microscopy

The samples were processed as previously described with some modifications (de Vivo et al. 2019; Nagai et al. 2021). Briefly, the infralimbic cortex was punched out using a 1.5‐mm trepan biopsy tool in the cryoprotectant solution. The punched‐out specimen was washed in the cutting solution for 3 min four times at 4°C, incubated for 1 h on ice with a solution of reduced osmium solution containing 1.5% potassium ferrocyanide, 2% osmium tetroxide, and 2 mM calcium chloride in 0.1 M cacodylate buffer, and exposed to a solution of 1% thiocarbohydrazide for 20 min at room temperature. The specimen was then incubated in 2% osmium tetroxide for 30 min at room temperature and incubated overnight with 1% uranyl acetate at 4°C. Then the specimen was stained with a solution of lead aspartate for 30 min at 60°C (pH 5.5) and dehydrated using ice‐cold solutions of freshly prepared 20%, 50%, 70%, 90%, 100%, and 100% anhydrous ethanol for 5 min each, followed by ice‐cold anhydrous acetone for 10 min twice. The specimens were then placed in the solution containing 50% Quetol 812 resin and 50% acetone for 45 min, and then 100% Quetol 812 resin for 45 min 3 times at 35°C. To increase electroconductivity of resin, we used carbon black filler, Ketjen black (Nguyen et al. 2016). Ketjen black was mixed with Quetol 812 resin in a 7:93 ratio. The specimen was flat embedded with ACLAR embedding film and kept in a 70°C oven for 72 h.

The resin‐embedded specimen was observed by Sigma scanning electron microscope (Carl Zeiss Microscopy, Goettingen, Germany) equipped with 3View technology (Gatan Inc., Pleasanton, CA, USA). In serial block‐face scanning electron microscopy (SBEM), serial images were obtained by scanning the face of an unsliced block of tissue placed inside the microscope, then cutting off ultrathin slices using an automated microtome within the instrument. The newly exposed surface of the sliced block was rescanned until a stack of images was obtained. Serial images were acquired using an aperture of 30 μm, high vacuum, acceleration voltage of 1.5 kV, image size of 5000 by 5000 pixels, and image resolution (xy plane) of 5 nm, while ultrathin sections were cut at a nominal thickness of 50 nm. A stack of ~500 images was acquired per mouse (15,625 μm^3^), in layer 2/3 of the infralimbic cortex. Images were Gaussian filtered and automatically aligned using the open‐source software Fiji.

For ultrastructural image analysis, regions densely filled with neuropils were cropped to a volume of 10 μm × 10 μm × 10 μm. From each image stack, 80 synapses were randomly selected and analyzed using the TrakEM2 plugin within Fiji software. The axon‐spine interface (ASI) area was quantified as a structural index of synapse size. To define this interface, dendritic spines were annotated, and the surface area in direct contact with the apposed axon forming a synapse was identified. The interface area was then calculated using Amira software (Thermo Fisher Scientific, Waltham, MA, USA). In addition to synapse size, several ultrastructural features were assessed as histological parameters. Specifically, the presence or absence of presynaptic mitochondria and the presence or absence of a spine apparatus within the dendritic spine were recorded. For a subset of ~30 randomly selected synapses per stack, the presence of tripartite synapses was evaluated by determining whether fine astrocytic processes were present at the perisynaptic region and made direct apposition to the synaptic cleft. In these synapses, astrocytic coverage was further assessed by tracing the ASI perimeter and the astrocyte‐apposed segment across serial sections, and the proportion of the ASI perimeter contacted by astrocytic processes was quantified based on previously established methods (Bellesi et al. 2015).

### 4.5 Proteomic Analysis

Proteomic sample preparation and analysis were performed according to established protocols with minor modifications (Kawashima et al. 2022). Samples were cleaned with cold acetone and incubated at −20°C for 2 h. After centrifugation, precipitates were dissolved in 100 mM Tris (pH 8.5) with 0.5% sodium dodecanoate using an ultrasonic homogenizer. Protein concentration was measured using a BCA assay and adjusted to 1 μg/μL. Disulfide bonds were reduced with 10 mM DTT at 50°C for 30 min, followed by cysteine alkylation with 30 mM iodoacetamide (IAA) in the dark at room temperature for 30 min. The reaction was quenched with 60 mM cysteine. For protein digestion, 150 μL of 50 mM ammonium bicarbonate was added, and proteins were digested overnight at 37°C with Lys‐C and trypsin (400 ng each). Peptides were acidified with 5% trifluoroacetic acid, centrifuged, desalted using a C18 spin column, dried, and reconstituted in 3% acetonitrile (ACN) with 0.1% formic acid. Peptide concentration was adjusted to 500 ng/μL.

For nanoLC‐MS/MS analysis, 500 ng of peptides were injected into an UltiMate 3000 RSLCnano LC System (Thermo Fisher Scientific) with an Aurora Series emitter column (75 μm × 250 mm) at 60°C. Solvents were 0.1% formic acid in water (A) and 0.1% formic acid in 80% ACN (B). The gradient was 2%–4% B for 0–3 min, 4%–34% B for 3–113 min, 34%–65% B for 113–118 min, and 65% B for 118–123 min at 400 nL/min for 0–3 min and 200 nL/min for 3–123 min. The Orbitrap Exploris 480 MS (Thermo Fisher Scientific) was used in ESI positive mode. Full MS1 scans (495–745 m/z) alternated with DIA MS2 scans (200–1800 m/z) using a resolution of 15,000 for MS1 and 45,000 for MS2, AGC target of 3e6, and isolation window of 4.0 m/z.

Data were analyzed using Scaffold DIA (Proteome Software) with the Mouse UniProtKB/Swiss‐Prot database. Parameters included HCD fragmentation, 10 ppm precursor and fragment tolerance, staggered DIA acquisition, trypsin digestion, 2–4 peptide charge, max one missed cleavage, and carbamidomethylation [C] as a fixed modification. Both peptide and protein FDR were set below 1%. Quantification results were then normalized to the median value of each sample. Proteins that were detected in only one sample were excluded from analysis.

The expression levels of proteins were represented as the signal intensities obtained from the nanoLC‐MS/MS system or as their Z‐score normalized value to enable the comparison of multiple proteins in the same analyses. To identify differentially‐expressed proteins depending on age (Age DEPs), a one‐way ANOVA was performed to compare the abundance of each protein across young, middle‐aged, and aged groups. Proteins with *p* < 0.05 were defined as Age DEPs. To identify proteins whose abundance correlated with cognitive performance in aged mice (Score CPs), Pearson correlation analyses were performed between protein abundance and behavioral accuracy among mice in the aged group; proteins with nominal (uncorrected) *p* < 0.05 were defined as Score CPs. To assess the robustness of these correlations, an exact permutation test was performed, evaluating all 8! = 40,320 possible permutations of the behavioral scores among the 8 aged mice, and Benjamini‐Hochberg FDR correction (*q* < 0.05) was applied to the parametric *p* values; because no protein survived FDR correction in either fraction, Score CPs were treated as exploratory candidates rather than statistically confirmed differentially expressed proteins. To evaluate whether this individual‐protein‐level limitation affected the overall biological conclusions, rank‐based gene set enrichment analysis (GSEA, version 4.4.0; Broad Institute) was performed using the GSEA_Preranked module, with all proteins ranked by their signed correlation coefficient with cognitive performance, without an individual‐protein significance threshold. Gene sets were obtained from the GO Biological Process 2023 collection (Enrichr), excluding gene sets containing fewer than 5 or more than 500 genes, and enrichment significance was assessed using 1000 gene‐set permutations, with FDR correction applied across all tested gene sets. Hierarchical clustering analyses were then performed using Age DEPs and Score CPs, and the major clusters were defined as C1 and C2 in Figure 2.MitoQ DEPs were defined as proteins with nominal *p* < 0.05 by unpaired two‐tailed *t*‐test between MitoQ‐ and dTPP‐treated aged mice.

Gene ontology (GO) analysis was performed on designated proteins using the publicly available resource at https://geneontology.org/. GO terms containing more than three and fewer than 500 assigned proteins were included in the analysis. Up to six out of the top 10 GO terms based on fold enrichment were displayed. When calculating log_2_ fold changes between groups, if a protein was undetected in one group, a placeholder value of +10 or −10 was assigned for visualization purposes.

Protein–protein interaction networks were constructed using Cytoscape software (Shannon et al. 2003), referencing interaction data from the STRING database (Szklarczyk et al. 2023). Proteins with a high number of connections (i.e., high degree centrality) within the network were defined as hub proteins. Proteins constituting respiratory chain complexes were identified using publicly available database (https://www.genenames.org/).

### 4.6 Statistical Analyses

Data are shown as means ± SEM. Statistical analyses were performed using Prism 10.6 software (GraphPad Software, San Diego, CA, USA). *p* values less than 0.05 were considered statistically significant. Comparisons between two groups were analyzed using an unpaired *t*‐test (Figures 1I–K,M,O,P,R,V,X and 4B,C,H,K,L). Comparisons among more than two groups were analyzed using one‐way ANOVA followed by Holm‐Sidak's post hoc multiple comparison tests (Figures 1C–E,G and 2F,I). Correlation analyses were performed by Pearson's correlation test (Figures 1O,T,Y, 2L,O, 3E,F,J,K, and 4E). Comparisons against a designated value were analyzed using a one‐sample *t*‐test (Figures 3J and 4F; compared with 0). See the figure legends for details on the statistical analyses used in the Figures S1–S3.

## Author Contributions

R.Y., H.N. and T.F. designed the study; R.Y., H.N., C.N., Y.Z., M.N., K.O., Y.K., N.O. and T.F. performed and analyzed the results; H.N. and T.F. wrote the manuscript.

## Funding

This study was supported in part by grants from AMED (JP25wm0625121 to H.N., JP24wm0425001, 25zf0127010, 25zf0127012, 25wm0625324 to T.F.), grants from JST Moonshot R&D (JPMJMS239F), Grant‐in‐Aid for Transformative Research Areas (23H04234 to T.F.) and Leading Initiative for Excellent Young Researchers (LEADER 23H04234 to H.N.) from the Ministry of Education, Culture, Sports, Science and Technology in Japan, Grants‐in‐Aid for Scientific Research (21H04812, 24K22086 to T.F., 20K07288, 23K06358, 26K09826 to H.N.) from the Japan Society for the Promotion of Science in Japan, the Cooperative Study Program of the National Institute for Physiological Sciences (H.N.) 22NIPS207, and research grants from the Uehara Memorial Foundation (H.N.), Japan Foundation for Applied Enzymology (H.N.), the KANAE Foundation for the Promotion of Medical Science (H.N.), SENSHIN Medical Research Foundation (H.N., T.F.), Meiji Yasuda Life Foundation of Health and Welfare (H.N.), Takeda Medical Research Foundation (H.N.), Daiichi Sankyo Foundation of Life Science (T.F.), SRF (T.F.), and the Kazato Foundation (H.N.).

## Conflicts of Interest

The authors declare no conflicts of interest.

## Supporting information


**Figure S1:** Female and male C57BL/6J mice show similar age‐related cognitive inflexibility, despite slower visual discrimination learning in aged females. Behavioral performance of young and aged, male and female C57BL/6J mice in the visual discrimination and response direction tests. For the visual discrimination test, the correct response rate averaged within each session throughout the experiment (A), over the first 12 sessions (B), and over the last 3 sessions (C), as well as the number of sessions required to reach the performance criterion (D), are shown. For the response direction test, the correct response rate within each trial throughout the experiment (E) and within the last session (F) are shown. Data are presented as mean ± SEM. Statistical analyses were performed using one‐way ANOVA with Holm‐Sidak post hoc multiple comparisons. *p < 0.05, **p < 0.01; ns, not significant. Four young female, four young male, eight aged female, and eight aged male C57BL/6J mice were used.
**Figure S2:** Rank‐based gene set enrichment analysis (GSEA) identifies significant pathway‐level enrichment among Score CP correlations despite the absence of individually significant proteins. (A) Gene Ontology Biological Process terms significantly enriched (FDR *q* < 0.05) among whole‐tissue Score CPs, ranked by the absolute value of the normalized enrichment score, identified by GSEA preranked analysis of all quantified whole‐tissue proteins, ranked by their signed Pearson correlation coefficient with cognitive performance in aged mice (*n* = 8), without an individual‐protein significance threshold. Dot size indicates gene set size; dot color indicates ‐log10(FDR *q*‐value). (B) Representative running enrichment plots for Oxidative Phosphorylation and Proton Motive Force‐Driven ATP Synthesis in whole tissue. (C) The top 15 Gene Ontology Biological Process terms significantly enriched (FDR *q* < 0.05) among synaptosomal Score CPs, ranked by the absolute value of the normalized enrichment score, using all quantified synaptosomal proteins. (D) Representative running enrichment plots for Mitochondrial Respiratory Chain Complex Assembly, Oxidative Phosphorylation, and Cytoplasmic Translation in synaptosomes. In (B) and (D), the green line indicates the running enrichment score; black vertical lines indicate the rank position of proteins belonging to the gene set; the lower panel shows the ranked list metric (Pearson correlation coefficient with cognitive performance) across all quantified proteins. GSEA was performed using GSEA v4.4.0 (Broad Institute) with 1000 permutations; *p* values and FDR *q*‐values were calculated using a permutation‐based procedure with correction for multiple comparisons across all tested gene sets.
**Figure S3:** Synaptosome preparations contain presynaptic and postsynaptic markers at comparable abundance and retain an appreciable glial contribution. Expression percentile (percentile rank among all quantified proteins) of established presynaptic (Syn1, Syn2, Syp, Snap25, Vamp2, Stx1a, Stx1b, Bsn, Pclo, Syt1, Rab3a; 11 protein species), postsynaptic (Dlg4, Homer1, Shank1, Shank2, Shank3, Gria1, Gria2, Grin1, Grin2a, Grin2b, Gphn; 11 protein species), astrocytic (Gfap, Aldh1l1, Slc1a2, Slc1a3, Aqp4, Glul, S100b; 7 protein species), microglial (Aif1, Itgam, Tmem119, P2ry12, Cd68; 5 protein species), and oligodendrocyte (Mbp, Mog, Mag, Plp1, Cnp; 5 protein species) marker proteins in whole‐tissue and synaptosome fractions. For each marker protein, the mean was calculated as the average across all individual mice (four young, five middle‐aged, and eight aged male C57BL/6J mice; *n* = 17) and used for percentile ranking. Data are presented as the mean ± SEM across marker proteins within each category. Statistical analyses were performed using a two‐way repeated‐measures ANOVA followed by Sidak's multiple comparisons test comparing whole‐tissue and synaptosome fractions within each category. *p < 0.05, ****p < 0.0001. Red, whole tissue; purple, synaptosome. Each dot represents an individual marker protein. Note that presynaptic and postsynaptic markers both ranked among the most abundant proteins in the synaptosome fraction, indicating that this preparation does not resolve presynaptic from postsynaptic compartments. Astrocytic and oligodendrocyte markers were significantly lower in the synaptosome than in the whole‐tissue fraction, but remained in the upper range of the proteome, indicating that the glial contribution is reduced but not eliminated. Synaptosome‐derived findings are therefore described as synapse‐associated throughout the manuscript.

**Data S1:** acel70716‐sup‐0001‐Supinfo1.xlsx.

**Data S2:** acel70716‐sup‐0002‐Supinfo2.xlsx.

**Data S3:** acel70716‐sup‐0003‐Supinfo3.xlsx.

**Data S4:** acel70716‐sup‐0004‐Supinfo4.xlsx.

## Data Availability

The datasets generated during and/or analyzed during the current study are available from the corresponding author on reasonable request.
