# Supplementary material for: Synaptic Mitochondrial Oxidative Stress Contributes to Individual Variability in Age‐Related Cognitive Inflexibility in Mice
Source: Aging Cell. 2026 Sep 14;25(9):e70716. doi: 10.1111/acel.70716 (PMC13572969; doi:10.1111/acel.70716)
Supplement: Supplementary file 1 [file ACEL-25-e70716-s002.docx]

**Supplementary Fig. 1 Female and male C57BL/6J mice show similar age-related cognitive inflexibility, despite slower visual discrimination learning in aged females.** Behavioral performance of young and aged, male and female C57BL/6J mice in the visual discrimination and response direction tests. For the visual discrimination test, the correct response rate averaged within each session throughout the experiment (A), over the first 12 sessions (B), and over the last 3 sessions (C), as well as the number of sessions required to reach the performance criterion (D), are shown. For the response direction test, the correct response rate within each trial throughout the experiment (E) and within the last session (F) are shown. Data are presented as mean ± SEM. Statistical analyses were performed using one-way ANOVA with Holm-Sidak post hoc multiple comparisons. **P* < 0.05, ***P* < 0.01; ns, not significant. Four young female, four young male, eight aged female, and eight aged male C57BL/6J mice were used.

**Supplementary Fig. 2. Rank-based gene set enrichment analysis (GSEA) identifies significant pathway-level enrichment among Score CP correlations despite the absence of individually significant proteins.** (A) Gene Ontology Biological Process terms significantly enriched (FDR q < 0.05) among whole-tissue Score CPs, ranked by the absolute value of the normalized enrichment score, identified by GSEA preranked analysis of all quantified whole-tissue proteins, ranked by their signed Pearson correlation coefficient with cognitive performance in aged mice (n = 8), without an individual-protein significance threshold. Dot size indicates gene set size; dot color indicates -log10(FDR q-value). (B) Representative running enrichment plots for Oxidative Phosphorylation and Proton Motive Force-Driven ATP Synthesis in whole tissue. (C) The top 15 Gene Ontology Biological Process terms significantly enriched (FDR q < 0.05) among synaptosomal Score CPs, ranked by the absolute value of the normalized enrichment score, using all quantified synaptosomal proteins. (D) Representative running enrichment plots for Mitochondrial Respiratory Chain Complex Assembly, Oxidative Phosphorylation, and Cytoplasmic Translation in synaptosomes. In (B) and (D), the green line indicates the running enrichment score; black vertical lines indicate the rank position of proteins belonging to the gene set; the lower panel shows the ranked list metric (Pearson correlation coefficient with cognitive performance) across all quantified proteins. GSEA was performed using GSEA v4.4.0 (Broad Institute) with 1,000 permutations; P values and FDR q-values were calculated using a permutation-based procedure with correction for multiple comparisons across all tested gene sets.

**Supplementary Fig. 3 Synaptosome preparations contain presynaptic and postsynaptic markers at comparable abundance and retain an appreciable glial contribution.** Expression percentile (percentile rank among all quantified proteins) of established presynaptic (Syn1, Syn2, Syp, Snap25, Vamp2, Stx1a, Stx1b, Bsn, Pclo, Syt1, Rab3a; 11 protein species), postsynaptic (Dlg4, Homer1, Shank1, Shank2, Shank3, Gria1, Gria2, Grin1, Grin2a, Grin2b, Gphn; 11 protein species), astrocytic (Gfap, Aldh1l1, Slc1a2, Slc1a3, Aqp4, Glul, S100b; 7 protein species), microglial (Aif1, Itgam, Tmem119, P2ry12, Cd68; 5 protein species), and oligodendrocyte (Mbp, Mog, Mag, Plp1, Cnp; 5 protein species) marker proteins in whole-tissue and synaptosome fractions. For each marker protein, the mean was calculated as the average across all individual mice (four young, five middle-aged, and eight aged male C57BL/6J mice; n = 17) and used for percentile ranking. Data are presented as the mean ± SEM across marker proteins within each category. Statistical analyses were performed using a two-way repeated-measures ANOVA followed by Sidak’s multiple comparisons test comparing whole-tissue and synaptosome fractions within each category. *P < 0.05, ****P < 0.0001. Red, whole tissue; purple, synaptosome. Each dot represents an individual marker protein. Note that presynaptic and postsynaptic markers both ranked among the most abundant proteins in the synaptosome fraction, indicating that this preparation does not resolve presynaptic from postsynaptic compartments. Astrocytic and oligodendrocyte markers were significantly lower in the synaptosome than in the whole-tissue fraction, but remained in the upper range of the proteome, indicating that the glial contribution is reduced but not eliminated. Synaptosome-derived findings are therefore described as synapse-associated throughout the manuscript.
